# Annexins: players of single cell wound healing and regeneration

**DOI:** 10.1080/19420889.2019.1676139

**Published:** 2019-10-13

**Authors:** Swantje Christin Häger, Jesper Nylandsted

**Affiliations:** a Membrane Integrity, Cell Death and Metabolism, Center for Autophagy, Recycling and Disease, Danish Cancer Society Research Center, Copenhagen, Denmark; b Department of Cellular and Molecular Medicine, Faculty of Health Sciences, University of Copenhagen, Copenhagen, Denmark

**Keywords:** Plasma membrane repair, annexins, initial repair, regeneration, cell membrane, wound healing

## Abstract

Cell life is defined by a thin 4 nm plasma membrane, which separates the interior of a cell from its environment. Thus, disruption of the plasma membrane poses a critical risk to cells, which requires immediate repair to avoid uncontrolled osmotic lysis and cell death. The initial repair response to stop the leakage usually occurs within 10–45 s and implicates Ca^2+^-activated phospholipid-binding proteins including annexins. We previously reported that annexin-induced curvature of lateral membrane around the hole plays an important role for immediate resealing of human cancer cells. Once the breach has been sealed, the cell often regenerates itself by removing the damaged membrane. This process, which also involves annexins includes excision and shedding of damaged membrane implicating the endosomal sorting complex required for transport (ESCRT) III and actin cytoskeleton remodeling. Hence, studies of cell membrane repair mechanisms should differentiate between the immediate repair response happening within seconds and the subsequent regeneration phase, which occurs in the order of minutes to hours after injury.

The plasma membrane of eukaryotic cells constitutes the “margin between life and death for individual cells”[1]. Moreover, the plasma membrane in combination with the cytoskeleton defines the cell shape, facilitates cell-cell contacts and can anchor cells to the surrounding extracellular matrix. It is therefore crucial that the plasma membrane is kept intact, both for cell survival and for the cell system as a whole to remain well-functioning [2]. Once the cell has healed, it faces the issue of regenerating damaged cellular structures including the area around the injured membrane.

Focused research within plasma membrane repair mechanisms started in the 1990s, which seems late giving the fundamental importance of this mechanism for cell viability [3]. However, the field is gaining increasing attention. This is mainly driven by the association of membrane repair with several diseases where the strongest link between repair deficiency and disease pathology is observed for muscular dystrophies that lead to gradual muscle wasting and weakening [4,5]. On the other hand, our findings reveal that cancer cells are a counterexample to repair-deficient diseases, since they appear to be more dependent on efficient plasma membrane repair to cope with the physical stress imposed on them due to their invasive behavior [6,7]. Importantly, novel biophysical techniques to better address plasma membrane repair mechanisms have been introduced lately and further promoted research in this field.

Membrane integrity is governed by several repair and regeneration mechanisms that are likely to be utilized in combination depending on the kind of injury imposed on the membrane. One important hallmark in the repair response is influx of Ca^2+^ through the membrane lesion due to a more than a thousandfold concentration gradient between the extracellular space and the cytoplasm [8]. Ca^2+^ influx triggers the rapid recruitment of repair proteins within 10–45 s to the injured membrane [9]. This initial recruitment of Ca^2+^-activated repair proteins such as annexins correlates with the time frame needed to seal a membrane hole to avoid excessive leakage of cell mass [10,11]. Thus, the damage response can be considered as a two-phase process: First, an immediate repair response to quickly seal the hole, and secondly – a regeneration phase to fully reestablish the membrane upon damage [12] (Figure 1). Although mechanisms of single-cell regeneration upon injury are poorly characterized these processes appear to depend on strategies to remove damaged membrane by endocytic mechanisms and by shedding of injured membrane.

**Figure 1.**
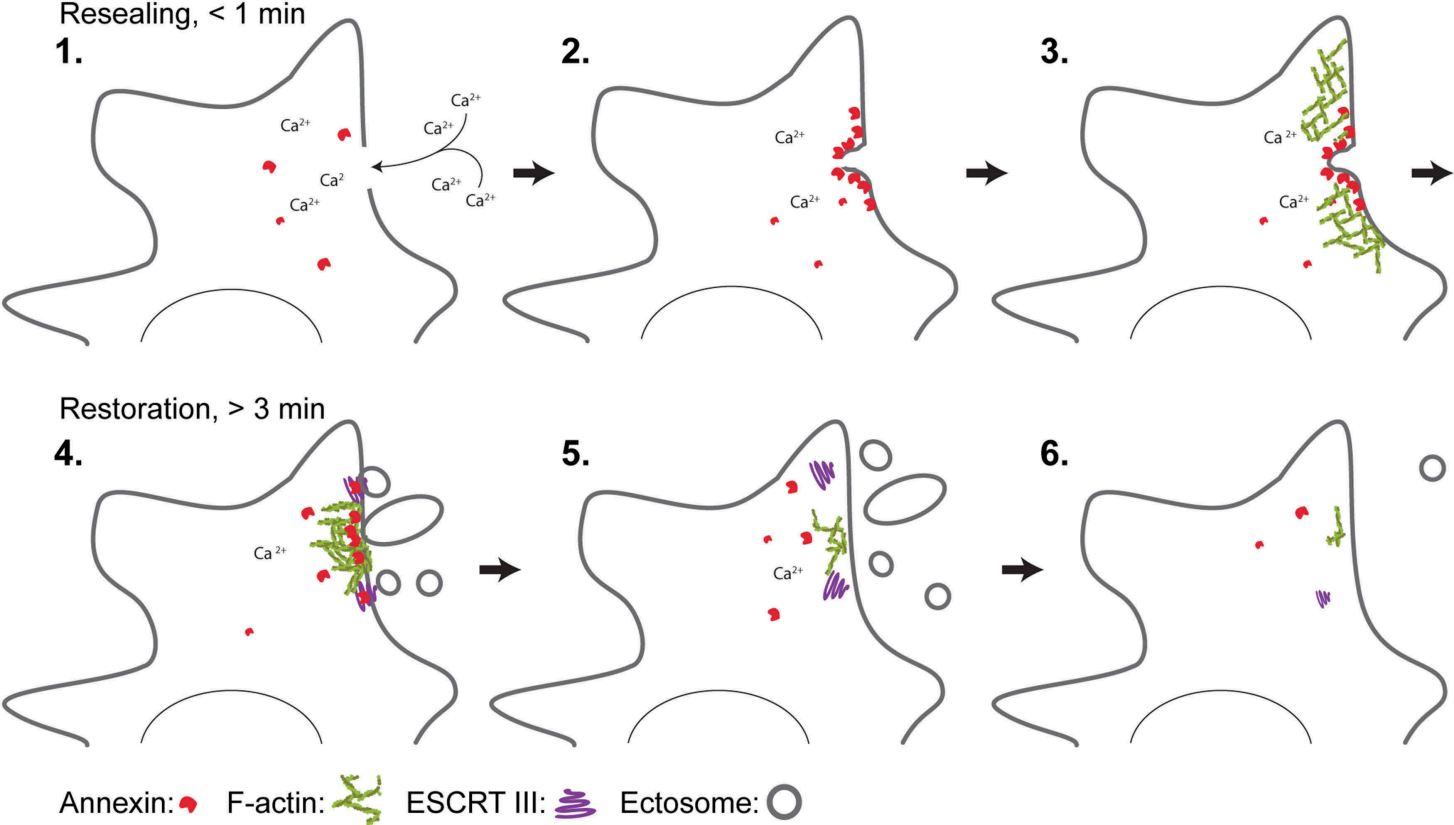
Model for plasma membrane repair in mammalian cells that discriminates between initial resealing and subsequent regeneration. Injury to the membrane and influx of Ca^2+^ ions into the cytoplasm activate annexin proteins that are recruited to the damaged membrane within 10-45 s. Here, ANXA4 and ANXA6 induce out-of-plane curvature and in-plane constriction, respectively, which in collaboration with other annexin family members enriches lateral membrane and promotes wound closure. Upon initial resealing, cells start to regenerate their membrane by shedding damaged membrane and/or by internalization (not shown). Excision of larger membrane areas requires F-actin buildup, which is regulated by ANXA2 protein at the injured membrane. Smaller membrane pieces are shedded in the form of ectosomes – a process that is facilitated by the ESCRT III complex. ANXA7 is needed to position and attach the ESCRT III components ALG-2 and ALG-2-interacting protein X (ALIX) at the injured membrane during this phase.

The cellular damage response can trigger the fusion of intracellular compartments with each other and the plasma membrane, which led to the patch hypothesis; it suggests that intracellular organelles fuse to form an impermeant patch at the damaged membrane [13]. While patching has been observed in large cell types such as eggs and oocytes of echinoderms [14] and amphibians [15] the process appears to be too slow alone to account for the rapid initial resealing (within 10–45 s) observed in many mammalian cell types. Moreover, we have not detected any patch formation during repair of human cancer cells such as HeLa cervix carcinoma and MCF7 breast carcinoma cells [6]. Our results support an alternative model in mammalian cells where lateral recruitment of membrane around the hole is used for resealing as reported from a mouse model of dysferlin-deficient muscular dystrophy [16]. Here, the membrane necessary for resealing is derived from the surrounding sarcolemma, enriched and then fused at the wound site [16]. In line with this, we have seen related repair responses implicating the manipulation of wound edges and lateral membrane in breast cancer cells upon wounding by ablation laser [17]. This response involves several annexin family members that upon injury to the plasma membrane and Ca^2+^ influx are rapidly recruited to the damaged membrane. To this end, we have assessed the impact of nine annexin family members (ANXA1-ANXA7, ANXA11, ANXA13) on supported membrane patches with free edges – a model system relevant for mimicking the condition around a membrane hole [18]. Interestingly, we found that a common characteristic of annexins is their ability to generate membrane curvature on anionic membranes, which seems to be important for their function during repair [18].

Upon plasma membrane injury, ANXA4 and ANXA6 are recruited to the damaged membrane and bind to and in the vicinity of wound edges. Here, self-association of ANXA4 into trimers induces out-of-plane curvature of membrane edges, whereas ANXA6 appears to trigger in-plane constriction of hole edges. The combination of these forces acts to pull the membrane edges together toward wound closure [17,19]. Moreover, we find that several other annexin family members bind around the injury site and may contribute by bending lateral membrane and glue adjacent membranes together [20]. Thus, the initial resealing response is likely orchestrated by annexin family members that are recruited to the injured membrane within the appropriate time frame (10–45 s) for rapid resealing. Once the initial leakage is stopped, the cell starts to regenerate and restructure the membrane often by removing the initially damaged membrane via shedding of damaged membrane and/or endocytosis (Figure 1). Some annexins are involved in this phase as well, and we have revealed that ANXA2 in a complex with S100A11 facilitates local cortical F-actin polymerization, which is required to excise the damaged part of the plasma membrane[6]. Since ANXA2 is recruited early, it probably serves a dual role by first facilitating curvature and fusion of lateral membrane at the wound site, and secondly to direct actin polymerization to restructure and excise the wounded part of the membrane.

Our recent data show that another annexin, ANXA7, which was the first annexin family member to be discovered [21], is involved in regenerating the plasma membrane upon damage [20]. Cells actively shed damaged membrane by ectocytosis through the endosomal sorting complex required for transport (ESCRT) III. The ESCRT III complex assembles spirally around the damaged membrane followed by contraction, which leads to shedding of membrane in the form of ectosomes. Here, the Ca^2+^-binding protein apoptosis-linked gene-2 (ALG-2) is needed to assemble the ESCRT III complex [22,23]. While ALG-2 lacks membrane-binding capability we found that ANXA7 is used to recruit and attach ALG-2 and ALG-2-interacting protein X (ALIX) to the damaged membrane. Thus, ANXA7 can initiate the process of ESCRT III buildup during the regeneration process to shed damaged membrane [20].

Single-cell membrane repair is an intriguing research field covering a wide panel of cellular processes, which need to be swiftly coordinated to reseal a torn membrane. However, how these processes are linked and regulated in detail is still unclear and requires further exploration. There seems to be some discrepancy between different cell studies, which may simply reflect general cellular plasticity, i.e. that different cell types repair differently. Thus, more interdisciplinary research combining biophysics with cell biology should provide better mechanistic insight into this fascinating repair response.
